# Supplementary material for: YPEL3 Negatively Regulates Endometrial Function via the Wnt/β-Catenin Pathways during Early Pregnancy in Goats
Source: Animals (Basel). 2022 Oct 28;12(21):2973. doi: 10.3390/ani12212973 (PMC9656084; doi:10.3390/ani12212973)
Supplement: Supplementary file 1 [file animals-12-02973-s001.zip › animals-1943313-supplementary.pdf]

Table S1. Primer pairs used for Real time quantitative PCR.

Name	Sequence of primer (5' to 3')	Literature or gene number
YPEL3-F	GCGGATTTCAAAGCCCAAGAC	XM_018040973.1
YPEL3-R	GGACTTGGAGATGAGGTCGT	
ISG15-F	GGTGAGGAACGACAAGGGTC	Yang et al. 2018
ISG15-R	CAGAATTGGTCCGCTTGCAC	
RSAD-F	TGCTTGGTGCCCGAGTCTAAC	Yang et al. 2018
RSAD-R	TCCGCCCATTTCTACAGTTCA	
CXCL10-F	AGGAATACACGCTGTACCTGC	NM_001285721.1
CXCL10-R	ACGTGGGCAGGATTGACTTG	
PGFS-F	TGGAGGACCCAGTTCTTTGTG	Yang et al. 2018
PGFS-R	TACCTGATAGCGAAGGGCAAC	
PTGS1-F	TCACAGTGCGTTCCAACCTTATC	Yang et al. 2018
PTGS1-R	ACGGAGGGCAGAATGCGAGTA	
PTGS2-F	GAGTGTAGGATTCGACCAGTAT	Yang et al. 2018
PTGS2-R	CCTTGAAGTGGGTAAGTATGTAG	
PTGES-F	CATCAAAATGTACGCGGTGGC	Yang et al. 2018
PTGES-R	GTCCTCGGGGTTGGCAAAAGC	
GAPDH-F	GATGGTGAAGGTCGGAGTGAAC	XM_005680968.3
GAPDH-R	GTCATTGATGGCAACGATGT	

Table S2. Short hairpin interfering RNA (shRNA) inserts.

shRNA	Sequence (5' to 3')
shYPEL3-1	GATCCGCAACCACATGATCAAAGACAATTCAAGAGATTGTCTTTGATCATGTGGTTGCTTTTTTG<br>AATTCAAAAAAGCAACCACATGATCAAAGACAATCTCTTGAATTGTCTTTGATCATGTGGTTGCG
shYPEL3-2	GATCCGTCAGGCCTACCTGGATGATTGTTCAAGAGACAATCATCCAGGTAGGCCTGACTTTTTTG<br>AATTCAAAAAAGTCAGGCCTACCTGGATGATTGTCTCTTGAACAATCATCCAGGTAGGCCTGACG
shN	GATCCTTCTCCGAACGTGTCACGTTTCAAGAGAACGTGACACGTTCGGAGAATTTTTTG<br>AATTCAAAAAATTCTCCGAACGTGTCACGTTCTCTTGAAACGTGACACGTTCGGAGAAG
